# Prognostic value of tumor markers in malignant melanoma

**DOI:** 10.1186/1878-5085-5-S1-A36

**Published:** 2014-02-11

**Authors:** Inka Treskova, Jindra Vrzalova, Radek Kucera, Ondrej Topolcan, Vladislav Treska

**Affiliations:** 1 Department of Surgery, Faculty of Medicine and University hospital in Pilsen, Charles University in Prague, Czech Republic; 2 Immunoanalytical Laboratory, Department of Nuclear Medicine, University Hospital in Pilsen, Czech Republic

## Scientific objectives

The incidence of melanomas has been increasing over the past decades. According to WHO statistics 132 000 melanoma skin cancers occur currently each year globally, as an ozone levels have been depleting, the next incidence increase of melanomas is estimated. The melanoma is highly metabolically active tumor that releases number of molecules. This study evaluated the dynamics of tumor markers and their relation to prognosis of melanoma patients.

## Technological approaches

In a group of 77 patients with malignant melanoma and cohort of 34 patients a control group, we have measured circulating levels of several biomarkers. TPS was measured using IRMA immunoassay (IDL Biotech, Sweden). S100A, OPN, OPG, IL-2, IL-6 and IGFBP-3 were measured using a novel multiplex xMAP technology on the instrument Luminex 100 (Merc, USA). Peripheral blood (20 mL) was drawn from each of the subjects using standardized phlebotomy procedures. The peripheral blood was drawn by VACUETTE ® (Greiner Bio-One, Austria) with and without EDTA as an anticoagulant. All specimens were immediately aliquoted and frozen, stored at -80°C. No more than one freeze-thaw cycle was allowed before an analysis. Samples were collected before surgery. SAS 9.2 software was used for all statistical analysis. The Wilcoxon test was used to compare distributions of values between melanoma group and the group with the healthy persons.

## Results interpretation

We found statistically significant elevated serum levels in melanoma group compared to healthy controls of S100A, TPS, OPN, IL-2, IL-6 and IGFBP-3. Elevated IL-6 and IL-10 preoperative serum levels in primary tumors were significantly associated with increasing of Breslow score. Elevated levels of S100A protein were positively correlated with Breslow score in advanced disease. In our study only higher serum levels of OPN and IL-2 demonstrated significant correlation with the presence of lymphatic node metastases.

## Outlook and Expert recommendations

Understanding the correlations between the prognostic factors and biology of the disease is a major objective of melanoma research. Certain biomarkers can reflect tumor mass, angiogenic potential or aggressiveness and allow a detailed categorization that is useful for prediction the biological activity of tumor. Monitoring of biomarkers improves estimation of prognosis, monitoring of patients and prevention of disease progression and individualization of treatment.

